# Investigation on Beam Alignment of a Microstrip-Line Butler Matrix and an SIW Butler Matrix for 5G Beamforming Antennas through RF-to-RF Wireless Sensing and 64-QAM Tests

**DOI:** 10.3390/s21206830

**Published:** 2021-10-14

**Authors:** Munsu Jeon, Yejune Seo, Junghyun Cho, Changhyeong Lee, Jiyeon Jang, Yejin Lee, Hyung-Wook Kwon, Sungtek Kahng

**Affiliations:** 1 Department of Information & Telecommunication Engineering, Incheon National University, Incheon 22012, Korea; Jeon@inu.ac.kr (M.J.); M.June@inu.ac.kr (Y.S.); elsa@inu.ac.kr (J.C.); fhtk02@inu.ac.kr (J.J.); yeeejjin@inu.ac.kr (Y.L.); 2 Convergence Research Center for Insect Vectors, Incheon National University, Incheon 22012, Korea; hwkwon@inu.ac.kr; 3 Center for Advanced Meta-Materials, Korea Institute of Machinery & Materials, Daejeon 34103, Korea; antman@kimm.re.kr

**Keywords:** millimeter-wave antenna, 5G antenna, beamforming antenna, Butler matrix, 64-QAM

## Abstract

In this paper, an intuitive approach to assessing advantages of beamforming in 5G wireless communication is proposed as a novel try and practical demonstration of importance of alignment between the transmitter’s and receiver’s beams working in millimeter-wave frequency bands. Since the diffraction loss of millimeter-wave signals matters seriously in propagation, the effects of the misalignment and alignment between beams need to be checked for, which was conducted with a horn antenna and the 4 × 4 Butler matrix which mimic the relationship of the base station and handset antennas. Designing and using the microstrip-line and the substrate integrated waveguide (SIW) Butler matrices, RF-to-RF wireless connectivity between the horn and the microstrip line beamformer as case 1 and the horn and the SIW beamformer as case 2, concerning the changing angle of the beam from either of the two Butler matrices, was tested, showing over 12 dB enhancement in received power. This direct electromagnetic link test was accompanied by examining 64-QAM constellations for beam-angle changing from −30° to +30° for the two cases, where the error vector magnitude in the QAM-diagram becomes less than 10% by beam-alignment for the changing angle.

## 1. Introduction

The 5th generation (5G) mobile communication is featured by technological fascination such as several Gbps data transfer-rate, low latency and low interference [1,2,3]. These three keywords can be attained by a macroscopic measure that the system architecture of the device is optimized; channel models are set up and monitored in real time; neighboring heterogeneous networks are found and connected with compatibility and adaptiveness; and a microscopic one that a wide-bandwidth of the millimeter-wave frequency is employed, and a wide-beam from the low-frequency handset is replaced by a narrow-beam from the high-frequency smartphone. Because the wireless signal should be emanated from the mobile device over the air and travels over the space on the net to the receiver, electromagnetic connectivity is very crucial. In order to realize the wireless connectivity for 5G, antennas operable in millimeter-wave frequency bands are needed. Making use of millimeter-wave antennas, the wide-bandwidth and narrow beamwidth would be accomplished by designing them to be arrays whose footprint is relatively small for even the commercial wireless phone. The beamwidth becomes narrow and pointy to have higher directionality in the far-field pattern. This is so-called beamforming. 

It is worthwhile to look over the kinds of beamforming antennas with feeding circuits in millimeter-wave bands and what they have suggested during the last couple of years. Y. Lee et al. showed an array antenna on the package driven by an RFIC [4]. The array elements are connected through multi-layers to the chip. That was intended for a handheld gadget, but Y. Kim et al. used a massive antenna loaded transmitter and a four-element mounted receiver to increase the data transmission-rate over 1 Gbps [5]. J. Park et al. saw the TX-to-RX link of antennas from the standpoint of a system and mentioned electromagnetic interference as a cause of degradation in function. W. Roh et al. shared the advantages of beamforming antennas enabling a mobile link to have a high throughput [6]. Y.-J. Cheng collected the reports on various kinds of millimeter-wave SIW circuits and SIW antennas, but their frequencies are away from 5G communication [7]. P. Arcioni et al. studied how appropriate an SIW is for the use in Ka-band [8]. Similarly, D. Liu took an example of the SIW used as the transition of a feed for a millimeter-wave antenna [9]. F. He et al. presented the positive outcome of the SIW in terms of integration into the planar circuits [10]. A very narrow beam at a fixed angle is generated by a slotted waveguide [11]. N. Ojaroudiparchin et al. designed a dipole and one pair of 1-by-8 array antennas [12]. Their radiating elements are metal on the stack of four RT substrates and placed on the platform with no enclosure. J. Park et al. showed a pair of LTCC (volumetric ceramic) array antennas located in the short side cover, opposed to the way the 5G mobile handset industry places antennas to face the wide back cover and long side cover [13]. As a sidenote, they wrongfully define their antennas as end-fire ones, although broadside antennas are obviously adopted. S. Chen et al. put a via-fence as a reflecting wall for a Yagi-dipole [14]. They are laid out on a platform without a cover. As to the single and array antennas which are exposed to the open space, five layers comprising RO4350B and RO4450F commonly used in academia. C. Di Paola et al. laid 5 Quasi-Yagi pointing to five different angles [15]. It can be classified as a space-diversity antenna. The antenna is formed as one layer of RO3003 as a truly simple structure in an open structure. W.-Y. Li et al. mimicked vertical via-array patch. W. Roh et al. mentioned above to make a 1-by-4 array on the side of the handset [16]. This LTCC antenna is added to a planar Quasi-Yagi array to work at two frequencies. They were treated with no barrier such as a housing. There is another move. How to maximize the usage and usefulness of relatively a limited area allowed for antennas in the platform is addressed. C. Lee et al. designed an SIW millimeter-wave antenna and added LTE-A MIMO antennas on the same metal plane [17]. They strategized reducing the footprints of low-frequency multiple antennas not to disrupt the 5G beamforming block. Similar to this in the coexistence of antennas for different services, W. Chung et al. put a folded loop antenna on the side by combining lines with vias and flanked by slot antennas [18]. This composite antenna is aimed at dual-band functions. In brief, all the work so far focused on component-level designs but did not handle the RF-to-RF wireless link. 

In this paper, a new way that 5G wireless system developers can obtain intuitions on the quality and effects of beamforming functions is suggested. This tried-and-true verification approach comprises the design of beamforming antennas and two experimental setups. Firstly, to give the capability of beamforming and beam-tilting to the wireless connectivity tests, the 4 × 4 Butler matrix was designed and manufactured as the microstrip-line structure and SIW structure. To enrich technical analysis and interpretation of this try, the two different antennas-under-test (AUT) were brought to the scene. Secondly, a measurement setup was devised to check RF-to-RF sensing between the horn and the microstrip Butler matrix as case 1 and between the horn and the SIW Butler matrix. By changing the angle of the beam from the Butler matrix in each case, the transmission coefficient of the beam from the horn to the RX beamforming antenna was recorded for beam misalignment and alignment. Thirdly, a setup was formed to watch 64-QAM diagrams for the two aforementioned cases. According to the change in the angle of the beam from either of the Butler matrices, 64-QAM constellations were plotted, and the misaligned beam in the wireless link resulted in very blurry pictures of digital symbol error spread. Notwithstanding, the beam alignment led to clear pictures of I/Q symbol spots. The tests revealed that beam alignment increases the received power by over 12 dB from the beam misalignment in the RF-to-RF and decreases the error vector magnitude to 10% or less in the QAM diagram.

## 2. Design of Two Planar Butler Matrix Antennas as Beamforming AUTs

### 2.1. The 4 × 4 Microstrip-Line Butler Matrix and Its Frequency Responses

The basic details of the Butler matrix are addressed in the Appendix A. Based on the design, beamforming antenna type 1 is physically realized as follows. 

**Figure 1:**
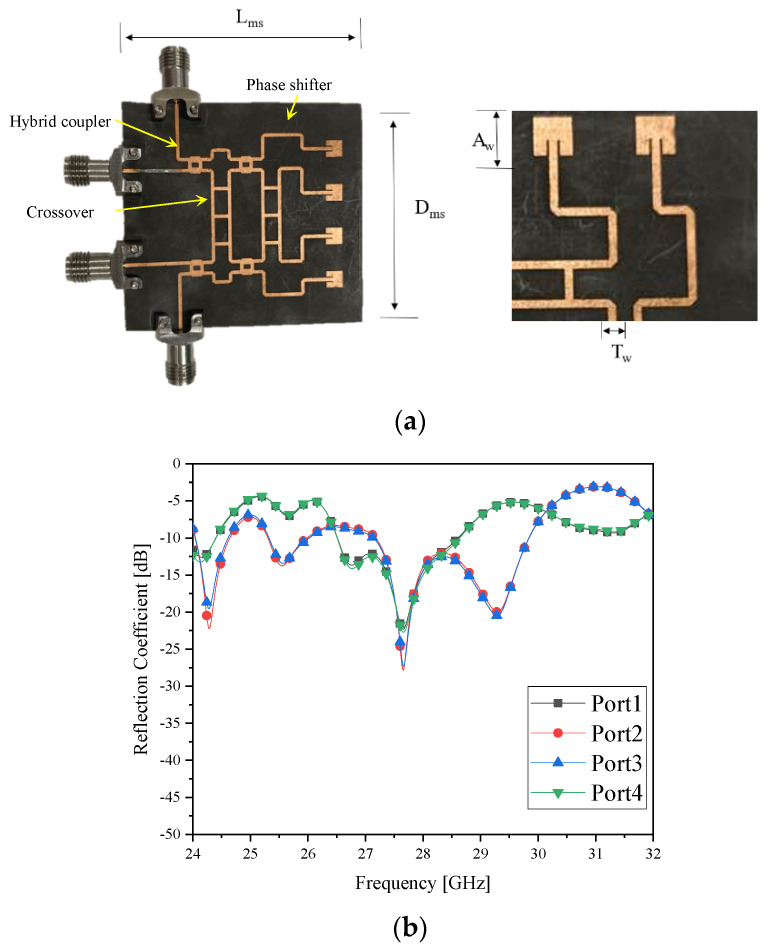

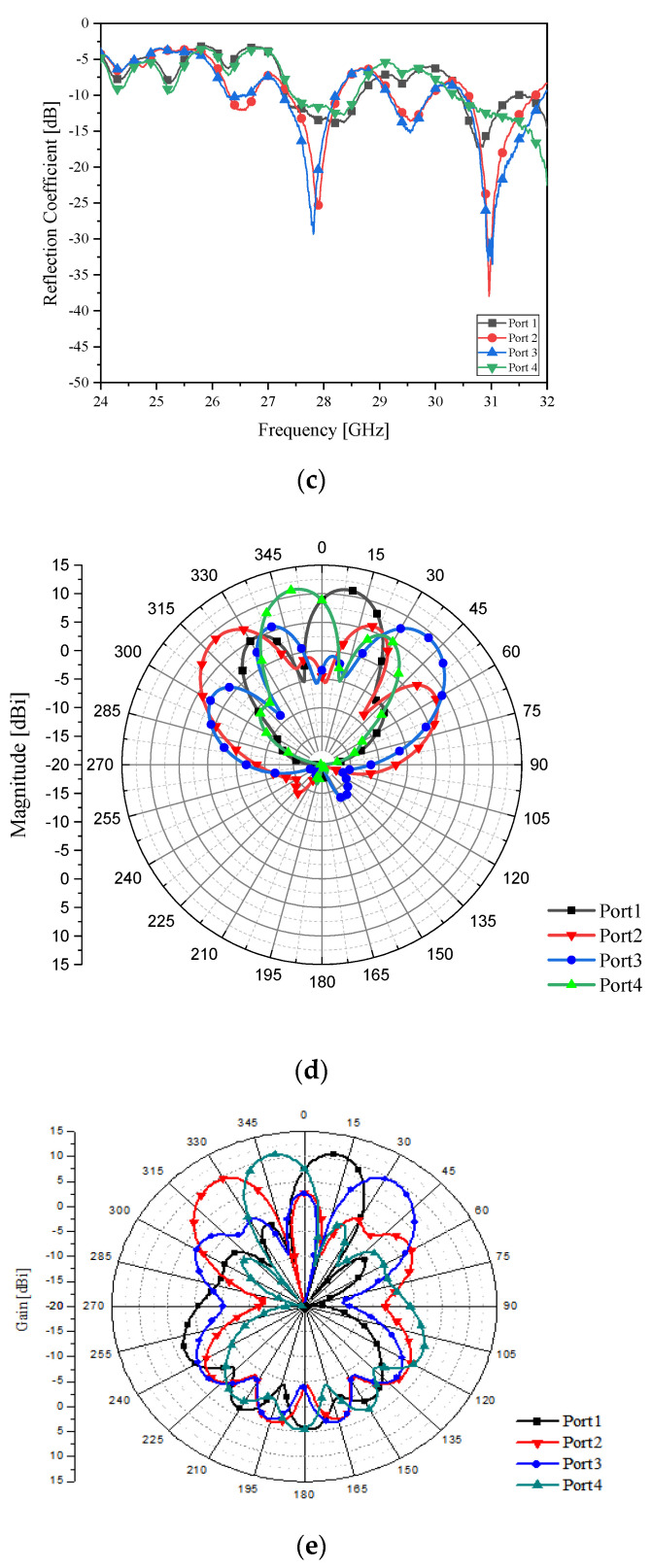
The microstrip-line Butler matrix (**a**) prototype; (**b**) port reflection coefficients(Sim.); (**c**) port reflection coefficients(Meas.); (**d**) beam-patterns(Sim.); and (**e**) beam-patterns (Meas.).

Figure 1a is the photograph of the prototype of the microstrip-line Butler matrix antenna looking similar to the one in [19]. This beamforming antenna has four input ports and four radiating elements at the output ports made on RT5880 as the substrate with thickness of 0.25 mm. The geometrical parameters noted in Figure 1a are mentioned in the followings as Table 1. 

Figure 1b,c are the reflection coefficients of the input ports of the antenna from the electromagnetic(EM) simulation and measurement. They present the impedance matching at 28 GHz as the 5G mobile frequency. The beamforming and beam-steering functions are observed in Figure 1d from EM simulation and Figure 1e from measurement. Once fabricated, the surface of the thin substrate tends to be bent and a little deformed due to the weight of the connectors, which causes differences, i.e., unwanted back radiation and a shift in the angles of the beams from the EM simulated data. The beams range from −30° to 30°, which is adopted to the change in the beam direction for RF-to-RF link tests and I/Q digital wireless evaluation.

### 2.2. The 4×4 SIW Butler Matrix and Its Frequency Responses

As seen previously, the area of the metalized part on the top-surface of the microstrip-line beamformer is even smaller than that of the dielectric part. This might be a cause of undesirable degradation concerned with unwanted electromagnetic radiation along the transmission-lines and unignorable leakage of RF signals to the air. This motivates antenna designers to choose the type of the structure that can suppress the unwanted energy-leakage as in the Appendix A. To cope with this negative phenomenon, the SIW beamformer is employed since it has metallic shields [17]. The densely populated vias along the sides of the guided-wave path imitate the metallic walls of the waveguide.

**Figure 2:**
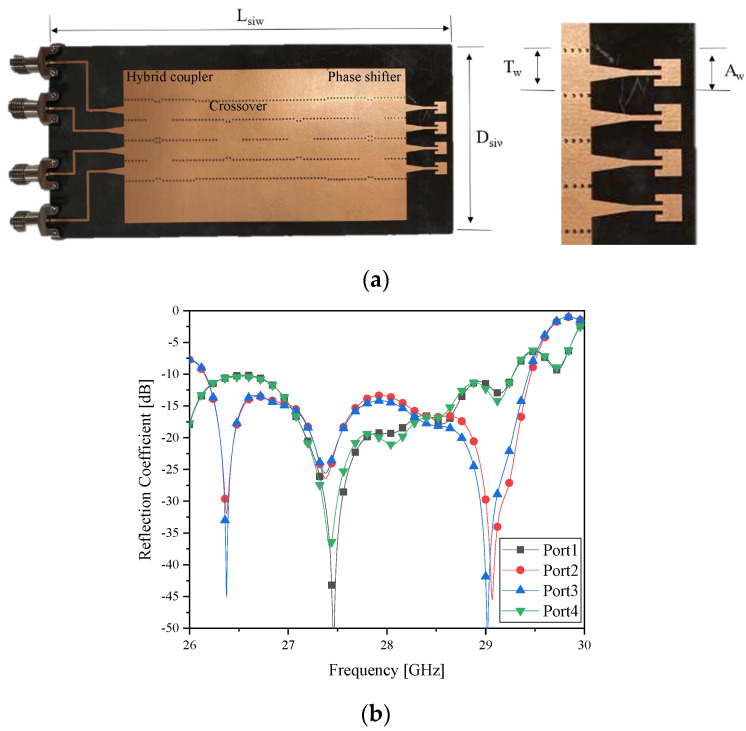

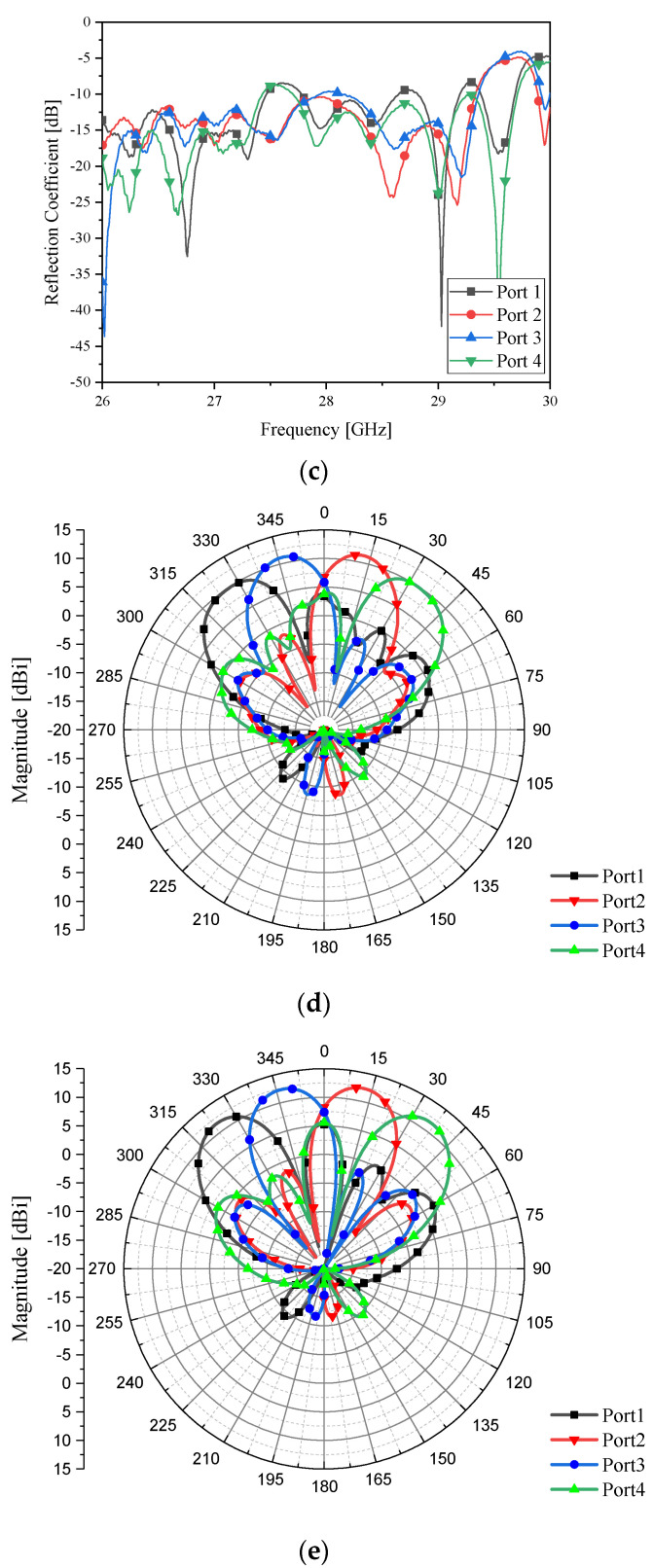
The SIW Butler matrix (**a**) prototype; (**b**) port reflection coefficients (Sim.); (**c**) port reflection coefficients (Meas.); (**d**) beam-patterns (Sim.); and (**e**) beam-patterns (Meas.).

Figure 2a displays the physically implemented SIW Butler matrix antenna. The metalized area is dominant on the top surface of the substrate in the contrary to the microstrip-line beamformer case. Because this aims at generating four beams, this antenna has four input ports and four output ports leading to the radiating elements. Its physical dimensions are given in Table 2.

Figure 2b,c are the simulated and measured reflection coefficients at input ports, implying the SIW beamformer works at 28 GHz. This input impedance matching turns out to have desirable beamforming and beam-tilting functions, as shown in Figure 2d as the simulated data and Figure 2e as the measured far-field patterns. The beam moves from −30° to 30° by selecting one out of the four input ports. Because of a good shielding property of the structure, the radiated field is better than Figure 1e.

## 3. RF-to-RF Connectivity Test and 64-QAM Investigation

As for a TX and an RX in 5G/6G mobile communication, there are four possible scenarios of beam pointing as in the following figure. 

**Figure 3:**
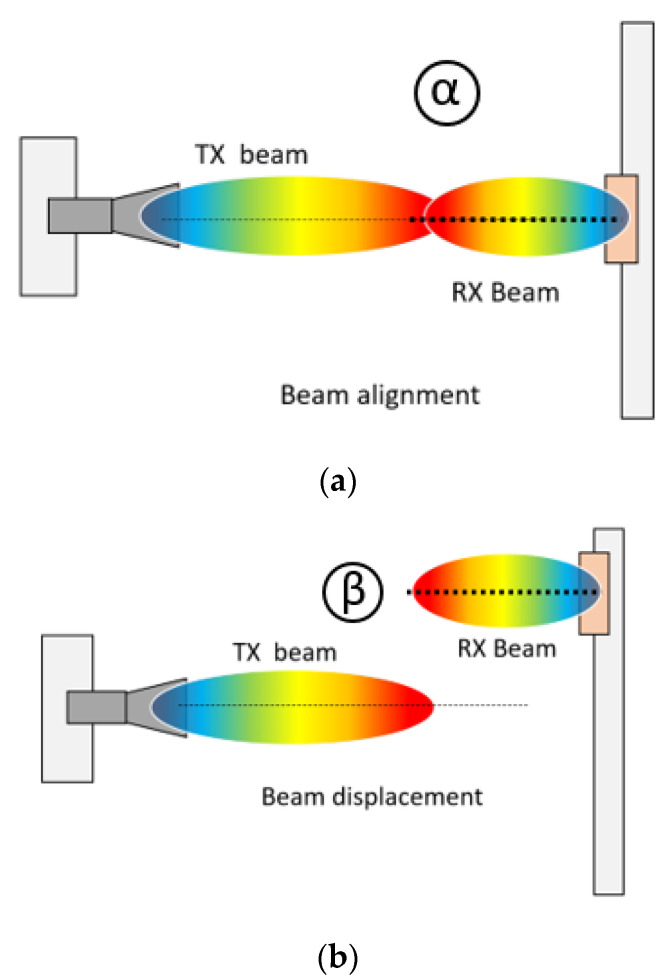

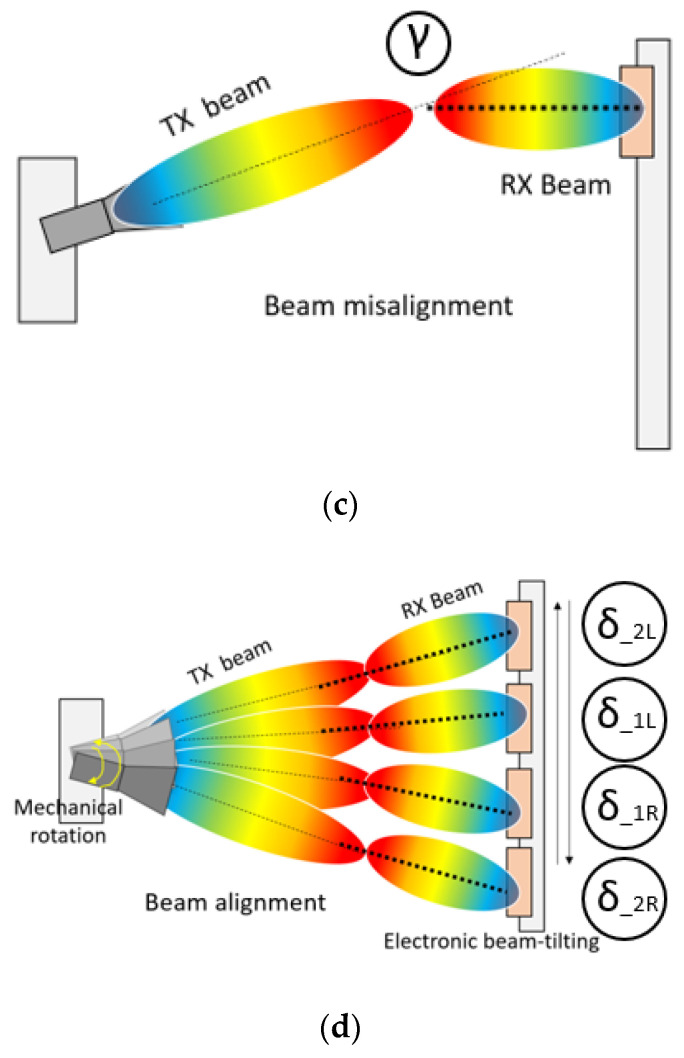
A variety of assumptions on directions of beams from the TX and RX antennas (**a**) in-line beam alignment; (**b**) beam displacement; (**c**) beam misalignment; and (**d**) beam alignment by cooperative TX and RX antennas.

Various situations of antenna positioning and beam pointing between the horn(TX) and the beamformer(RX) are represented by Figure 3. The strongest RF link is expected in Figure 3a as the in-line beam alignment (*α*). Located at different heights (*β*), though the TX beam is in parallel with the RX beam, this displacement degrades the wireless link as in Figure 3b. The beam misalignment ends up with the worst RF connectivity as in Figure 3c, denoted as (γ). As the location of the RX changes, the beam tilted by the RX catches the beam by the rotated TX horn, which means beam alignment, and results in much improved connectivity as in Figure 3d, denoted as *δ__2R_*, *δ__1R_*, *δ__1LR_* and *δ__2L_*.

**Figure 4:**
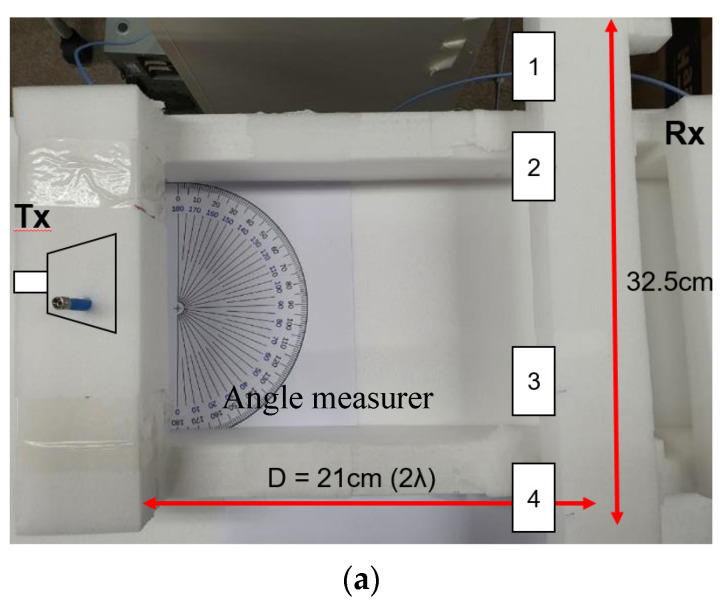

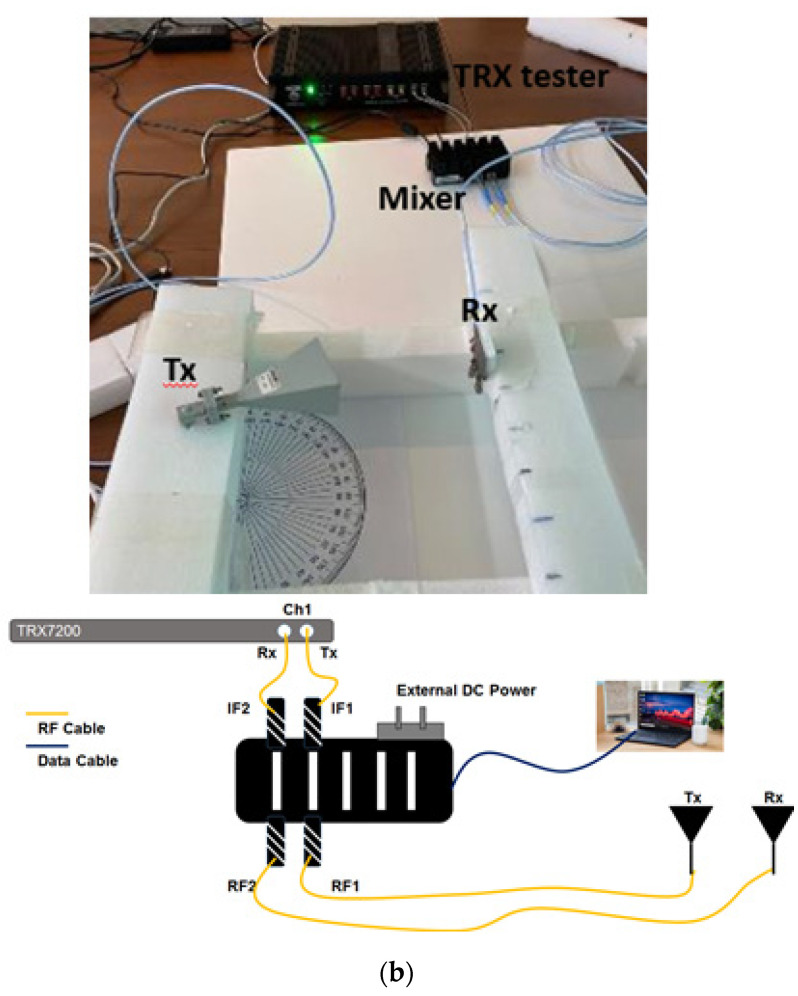
Two intuitive measurement configurations for checking 5G wireless connectivity enabled by antennas (**a**) RF-to-RF connection test with the vector network analyzer (**b**) I/Q communication and QAM observation test with TRX7200 and its companion up/down convertor.

Considering the RF-to-RF connectivity tests, Figure 4a marks the positions of the TX and RX antennas 21 cm apart or farther, assuming the gap as the far-field distance with respect to 28 GHz. The two sides are connected to the VNA ports. To relate the quality of 5G mobile communication to the beam-tilting capability and beam alignment of the antennas, QAM constellations are observed by equipment named TRX7200 and its mixer instead of the VNA as in Figure 4b. Both the measuring systems are free from the external amplifiers. In the first place, case *α* is conducted as the general reference.

**Figure 5:**
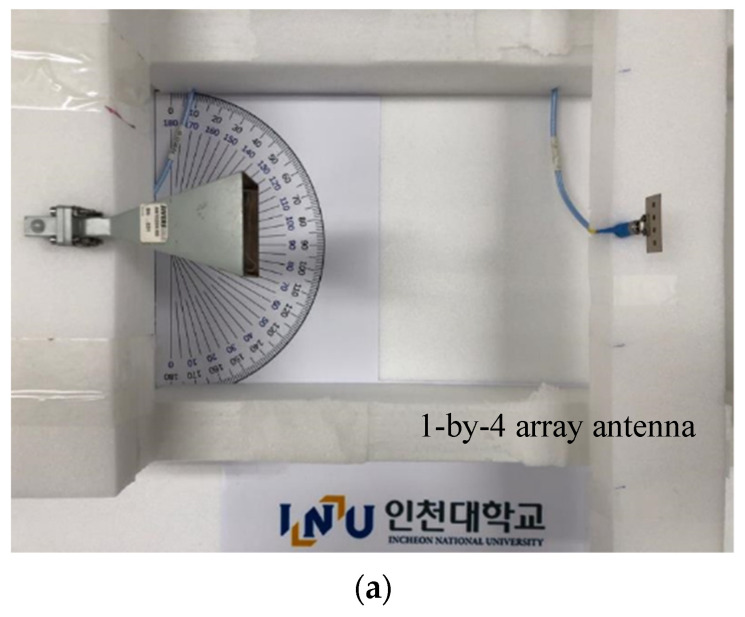

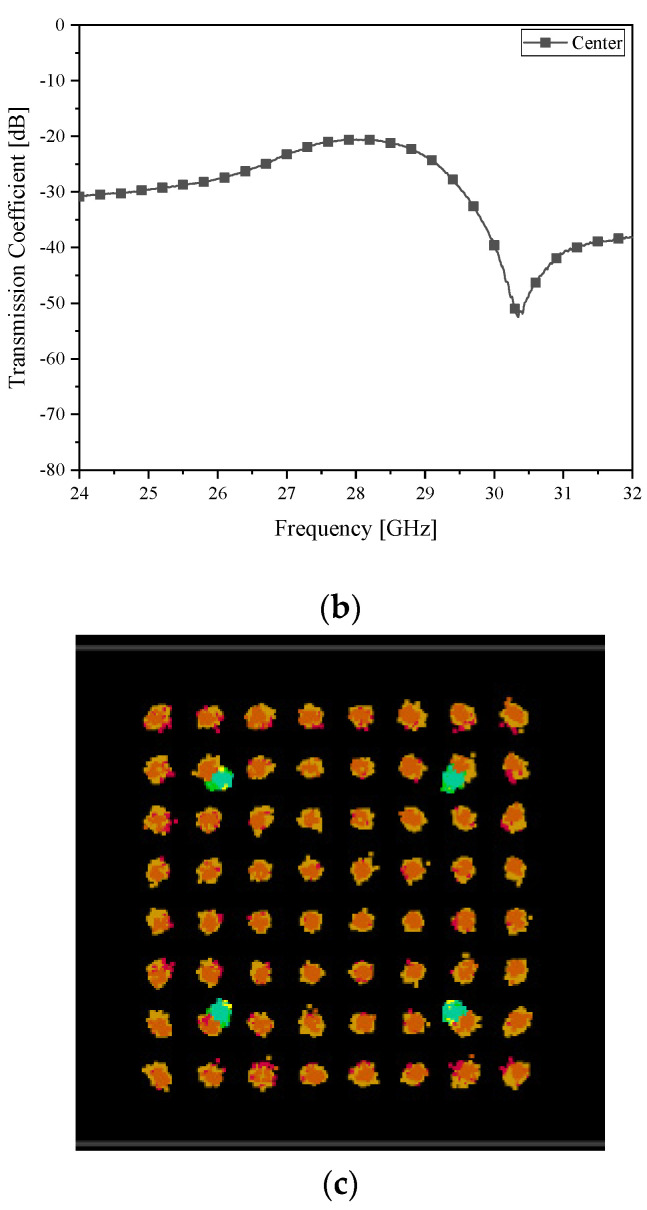
In-line beam alignment test as case *α* (**a**) RF-to-RF connection test; (**b**) transmission coefficient (strongest connectivity); and (**c**) I/Q Constellation (clear).

The horn antenna is laid in line with a 1 × 4 array antenna denoted as case *α*, and the received power of −19 dB is detected as S_21_ as in Figure 5a,b. The boresight beam from the RX accepts the RF signal from the TX as the strongest magnitude. Accordingly, its 64-QAM constellation is plotted as a clear picture. 

**Figure 6:**
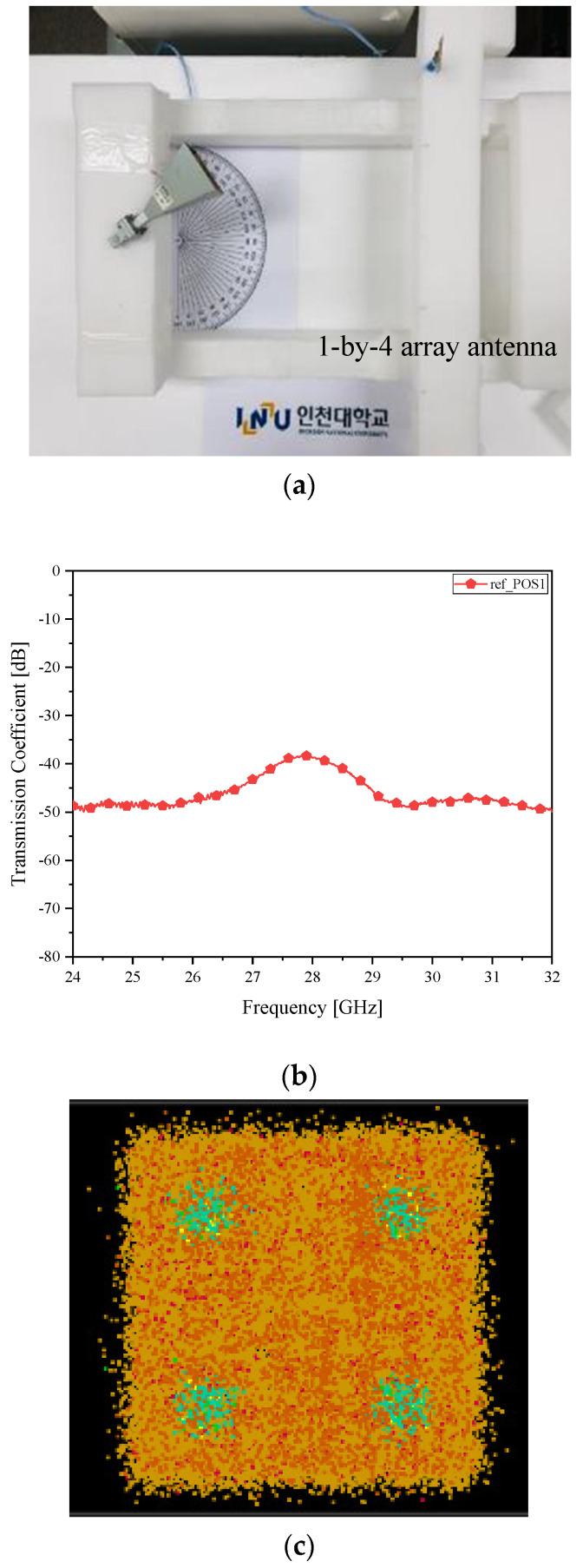
Beam misalignment verification as case *γ* (**a**) RF-to-RF connection test; (**b**) transmission coefficient (worst in connectivity); and (**c**) I/Q Constellation (most blurry).

The RX 1 × 4 array antenna with the boresight radiated field is relocated from the center of the left side of the test jig to position *δ__2L_*, and the RX horn antenna is mechanically rotated by 30° upward to move its beam as in Figure 6a. At first sight, the TX and RX antennas seem to see each other straight, and this might produce an acceptable level of received power. However, as is depicted in the case *γ* of Figure 3, the directions of the RX and TX beams differ from each other by 30° as the misalignment case, and the received power level drops by 20 dB, which is a serious degradation in the connectivity as in Figure 6b. This ends up with tremendous disruption in the QAM constellation in Figure 6c as the worst in wireless communication. Therefore, the RX should be replaced by one from the beamformers designed in Section 2.1 and Section 2.2.

**Figure 7:**
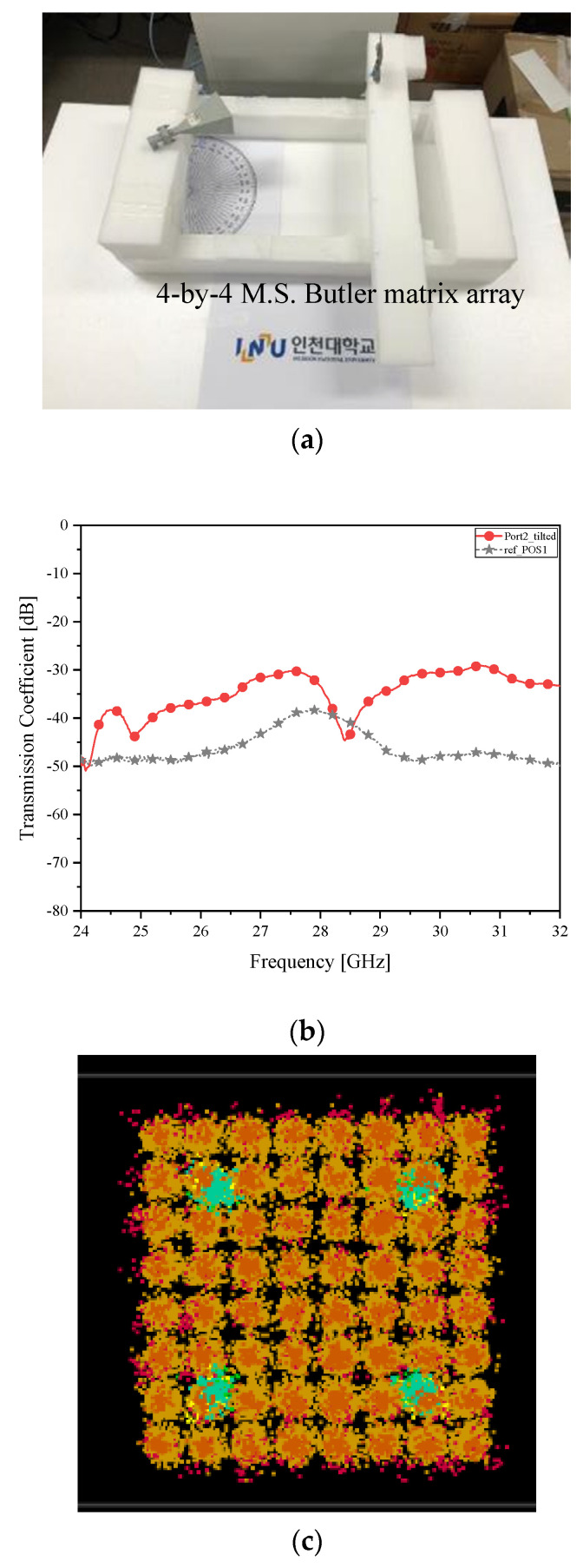
Beam alignment verification as case *δ__2L_* with the microstrip Butler matrix as the RX antenna (**a**) RF-to-RF connection test; (**b**) transmission coefficient (improved connectivity); and (**c**) I/Q Constellation (Clear).

The RX is situated at 30° from the center-line of the TX. In the previous test, despite the mechanical rotation of the horn, since the RX has no beam-tilting function, the two sides lose electromagnetic link. The microstrip-line Butler matrix antenna is substituted for the boresight antenna with a fixed beam. In addition, as case *δ__2L_*, the −30° tilted beam is radiated to the TX horn antenna as in Figure 7a, and S_21_ becomes −30 dB in Figure 7b where RF power transfer of the beamformers’ beam in red is stronger than that of the non-tilting beam in gray. The increment in RF-to-RF connectivity is led to enhancement in wireless communication. The poor performance in the Figure 6c is mitigated to the clear distribution of I/Q symbols as in Figure 7c.

**Figure 8:**
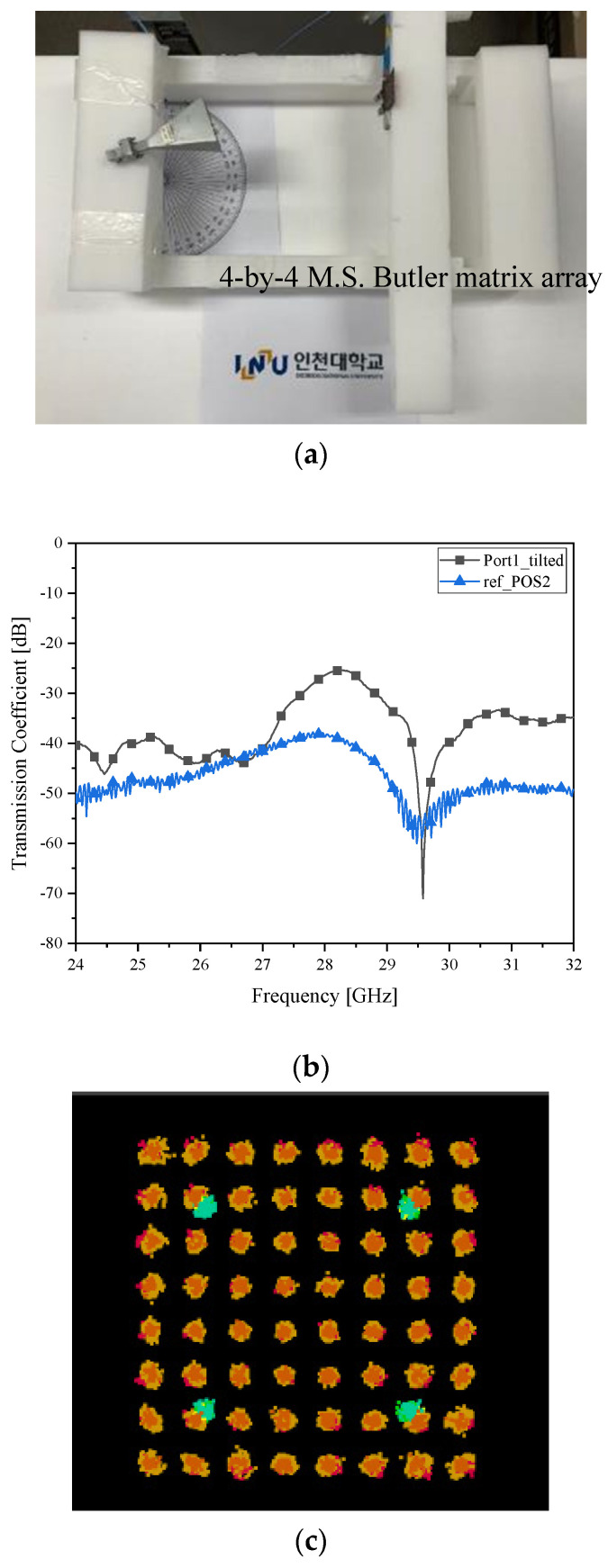
Beam alignment verification as case *δ__1L_* with the microstrip Butler matrix as the RX antenna (**a**) RF-to-RF connection test; (**b**) transmission coefficient (improved connectivity); (**c**) I/Q Constellation (very clear).

The RX is now located at 15° from the center-line of the TX. In the previous test, despite the mechanical rotation of the horn, since the RX has no beam-tilting function, the two sides are electromagnetically disconnected. The microstrip-line Butler matrix antenna is substituted for the boresight fixed beam antenna. In addition, as case *δ__1L_*, the −15° tilted beam is launched to the TX horn antenna as in Figure 8a, and S_21_ becomes −22 dB in Figure 8b. While the non-tilting antenna at the same position has −40 dB in S_21_, the beamformer’s received power is 18 dB higher. The increment in RF-to-RF connectivity leads to the enhancement in wireless communication. The pollution in the Figure 6c is mitigated by the clear distribution of I/Q symbols as in Figure 8c. Next is what becomes of the RX relocated to the angle of −15° on the right side of the test fixture. 

**Figure 9:**
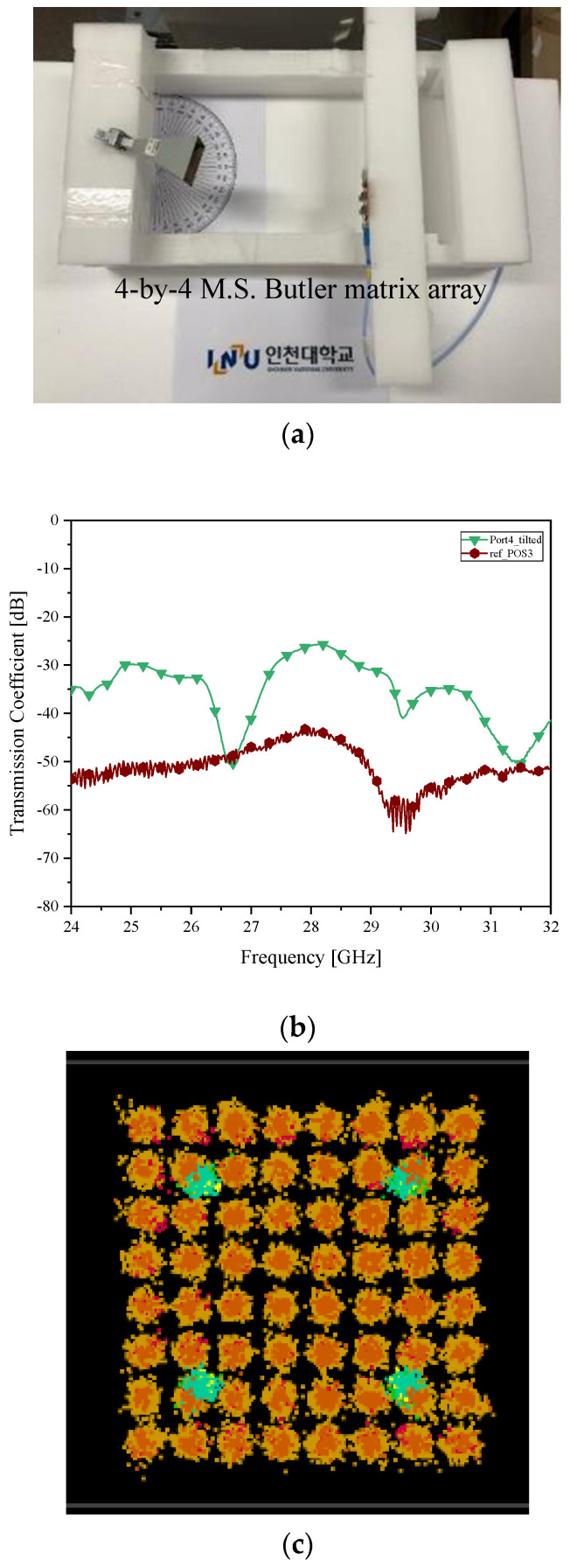
Beam alignment verification as case *δ__1R_* with the microstrip Butler matrix as the RX antenna (**a**) RF-to-RF connection test; (**b**) transmission coefficient (improved connectivity); and (**c**) I/Q Constellation (clear).

The angle between the positions of the RX and the center-line of the TX is 15° to the south of the area, and Figure 9a expresses that the 15° -angle pointed beam should emanate from the microstrip beamformer. Case *δ__1R_* beam from the RX is aligned with the −15° -angle inclined beam of the TX, which becomes the curve S_21_ in Figure 9b, which proves the beam alignment strengthens electromagnetic connectivity. Similar to Figure 8, this alignment between the wave-propagation directions is evident by the QAM constellations in Figure 9c. 

**Figure 10:**
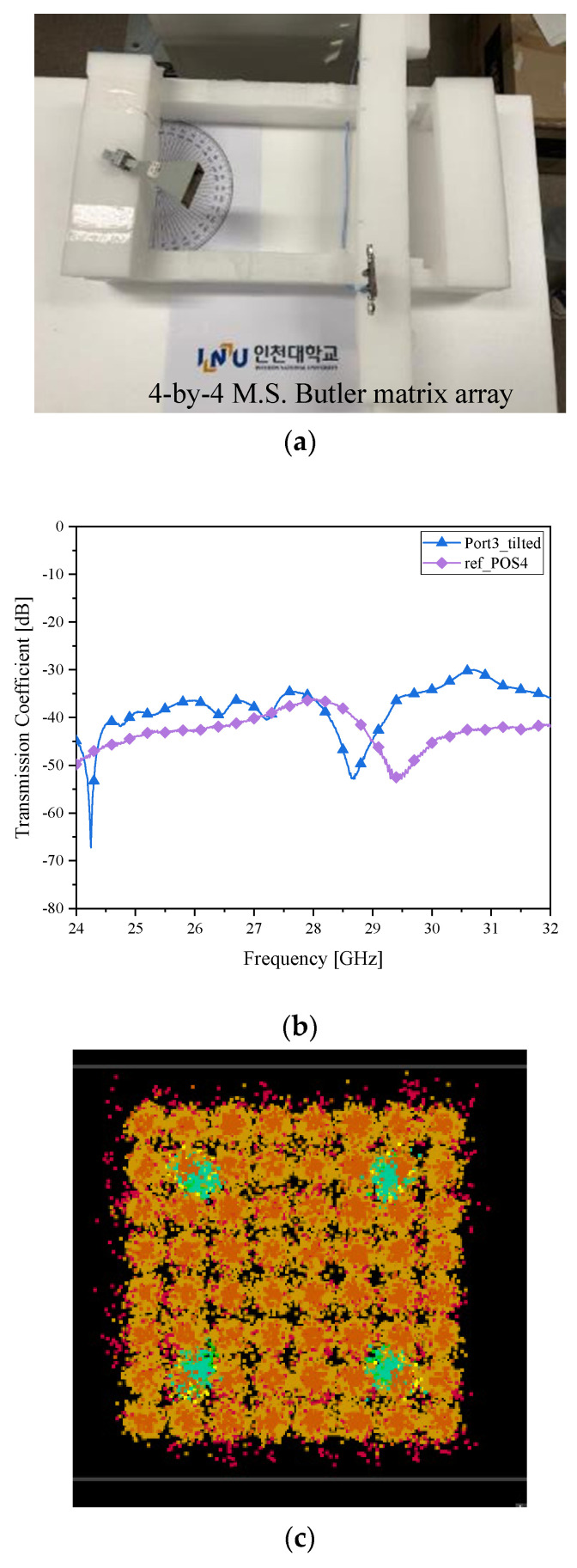
Beam alignment verification as case *δ__2R_* with the microstrip Butler matrix as the RX antenna (**a**) RF-to-RF connection test; (**b**) transmission coefficient (improved connectivity); and (**c**) I/Q Constellation (less clear).

As the position of the RX antenna is moved further south, the angle between the positions of the RX and the center-line of the TX is 30° in Figure 10a, that is to say, −30° as case *δ__2R_*. When the angle grows from 15 to 30°, the transmission coefficient as an indicator of magnitude of electromagnetic connectivity becomes lower, for the antenna gain of the more tilted beam becomes lower as explained in Reference 17. This is shown by Figure 10b, that there is almost no difference between two curves of S_21_ whether it is a tilted beam or not. This mediocre result is attributed to the leakage of the 28-GHz guided-wave in the microstrip-line structure, bent surface, etc. A new beamforming antenna is tried to tackle the problem of this undesirable phenomenon. 

**Figure 11:**
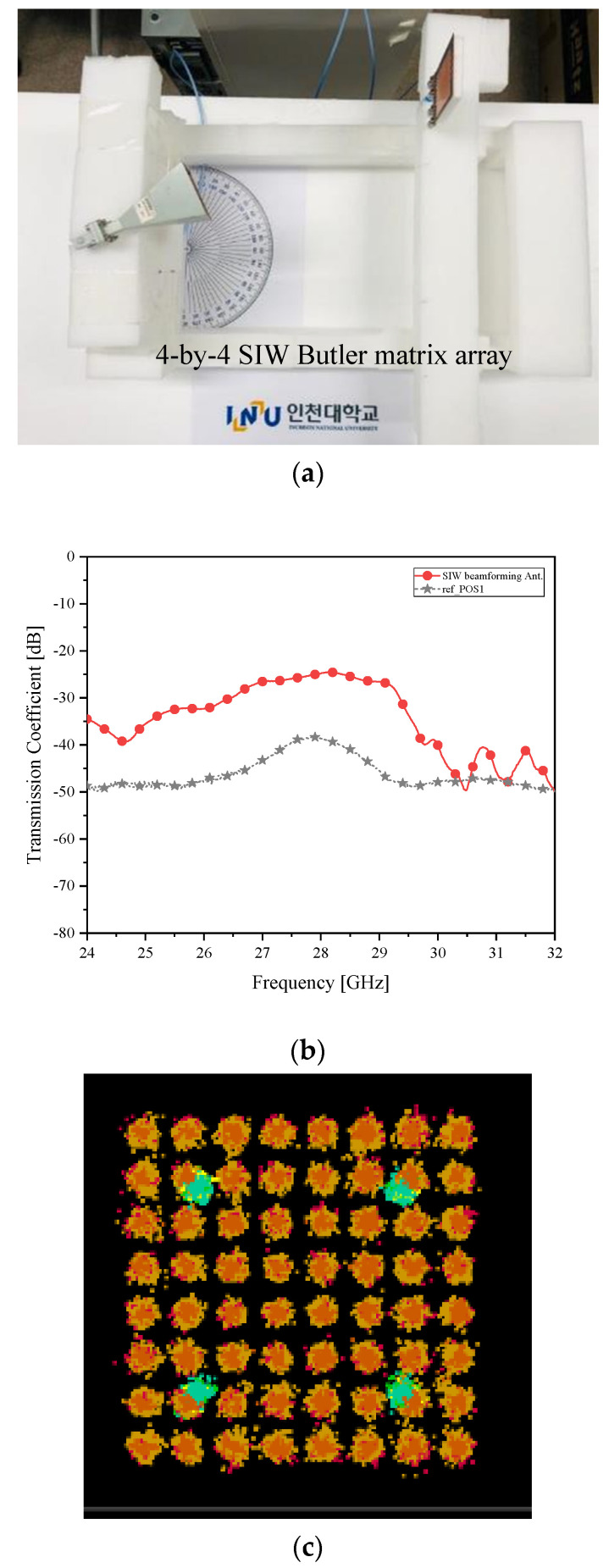
Beam alignment verification as case *δ__2L_* with the SIW Butler matrix as the RX antenna (**a**) RF-to-RF connection test; (**b**) transmission coefficient (Improved connectivity); and (**c**) I/Q Constellation (Clearer than the microstrip-line counterpart).

Figure 7 is with the microstrip-line Butler matrix antenna, but Figure 11a sets the SIW Butler matrix one for the RX. Because the cases of the farthest angles created by a 4-by-4 Butler matrix might give awkward electromagnetic linkage between the RX and TX and the EVM over 10% in the QAM test concerning the microstrip-line structure, there is an expectation on this new antenna. As for case *δ__2L_* in Figure 11b, the SIW beamformer raises the received power to −25 dB, a lot higher than Figure 7b and Figure 10b. This affects the 64-QAM test positively. As a result, the constellation in Figure 11c is clear.

**Figure 12:**
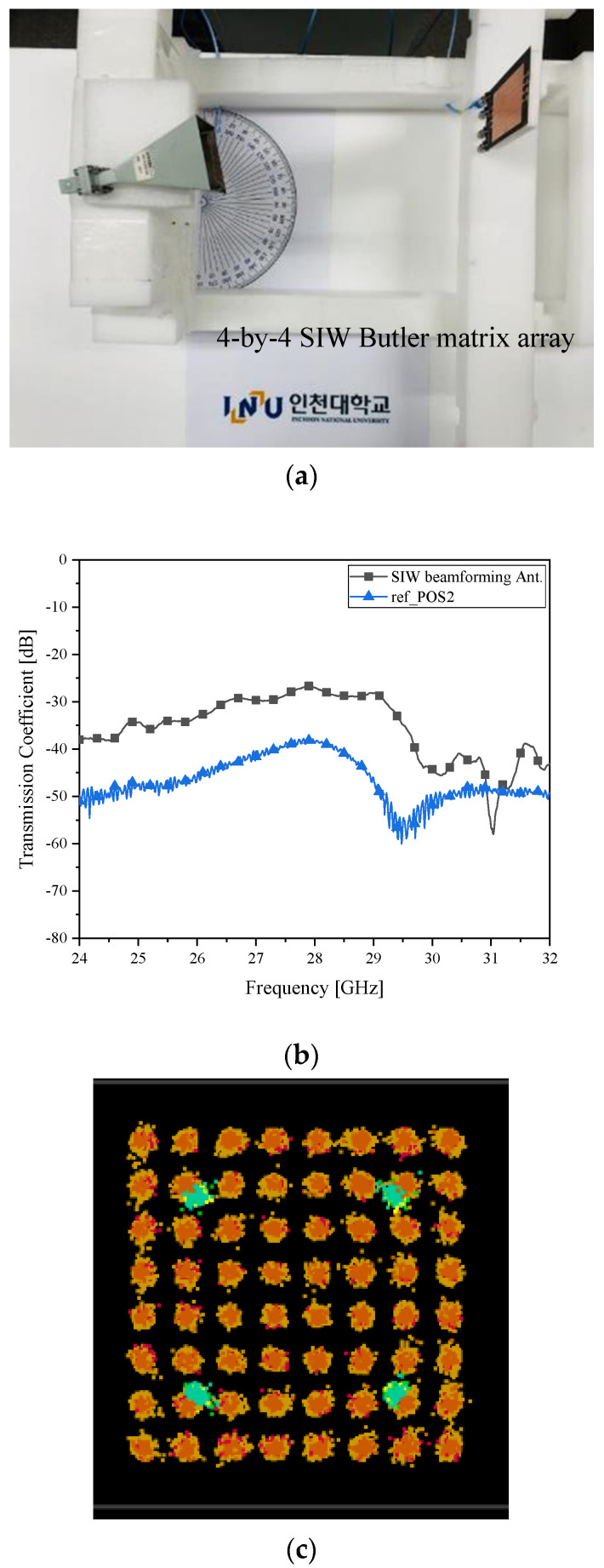
Beam alignment verification as case *δ__1L_* with the SIW Butler matrix as the RX antenna (**a**) RF-to-RF connection test; (**b**) transmission coefficient (improved connectivity); (**c**) I/Q Constellation (clear).

Figure 12a puts the position of the RX down by a notch. As case *δ__1L_*, the angle to the receiver is +15°. In Figure 12b, the transmission coefficient becomes −27 dB, which is as good as the level in Figure 8b which is the best of the four tested cases with the microstrip-line. As in Figure 12c, 64 I/Q symbols are distinct. 

**Figure 13:**
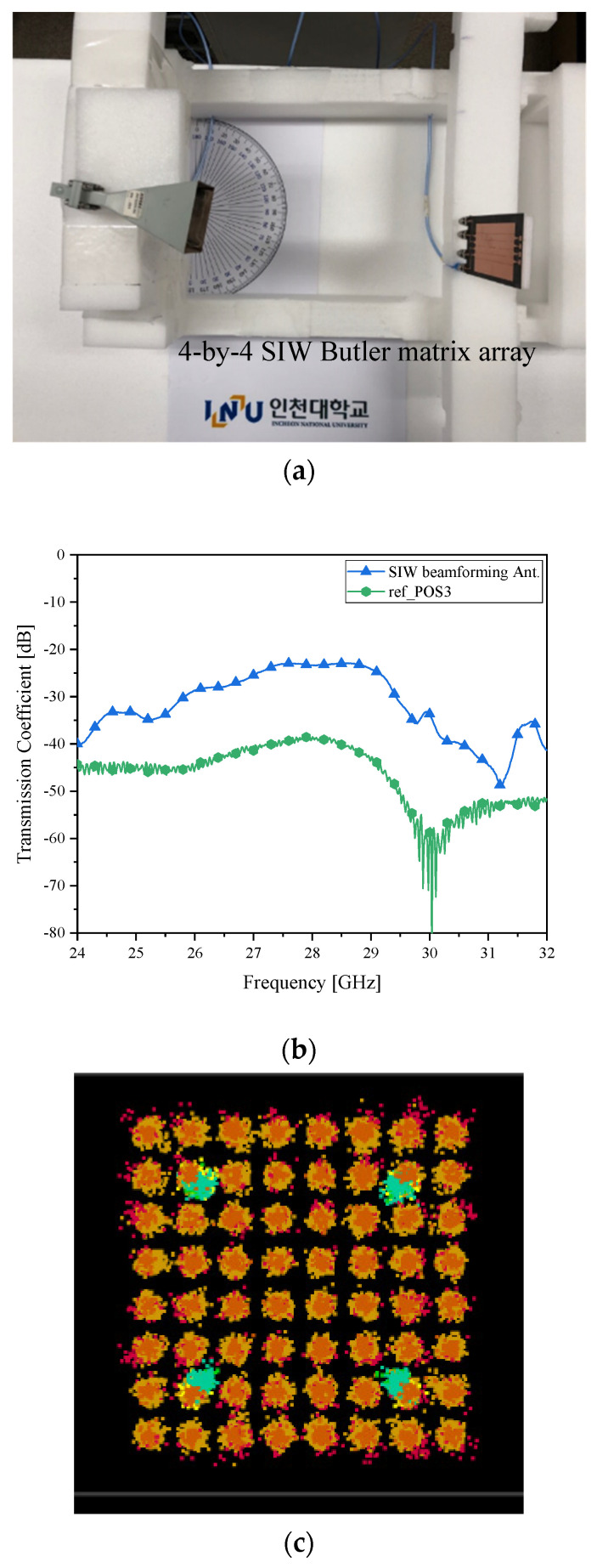
Beam alignment verification as case *δ__1R_* with the SIW Butler matrix as the RX antenna (**a**) RF-to-RF connection test; (**b**) transmission coefficient (improved connectivity); (**c**) I/Q Constellation (clear).

Figure 13a shows the RX is placed at the angle of −15°. Case *δ__1R_* looks symmetric with reference to Figure 12a. The +15°-tilted beam from the RX SIW Butler matrix connects electromagnetically to the TX horn for beam alignment, and this results in S_21_ of nearly −20 dB enhanced by 5 dB compared to case *δ__2L_* as presented in Figure 13b. Observing the data, the 1L and 1R beams emanated from the SIW Butler matrix have relatively a high antenna gain, which is helpful for building good electromagnetic linkage between the TX and RX. A clear constellation is achieved in Figure 13c due to the good performance shown in Figure 13b.

**Figure 14:**
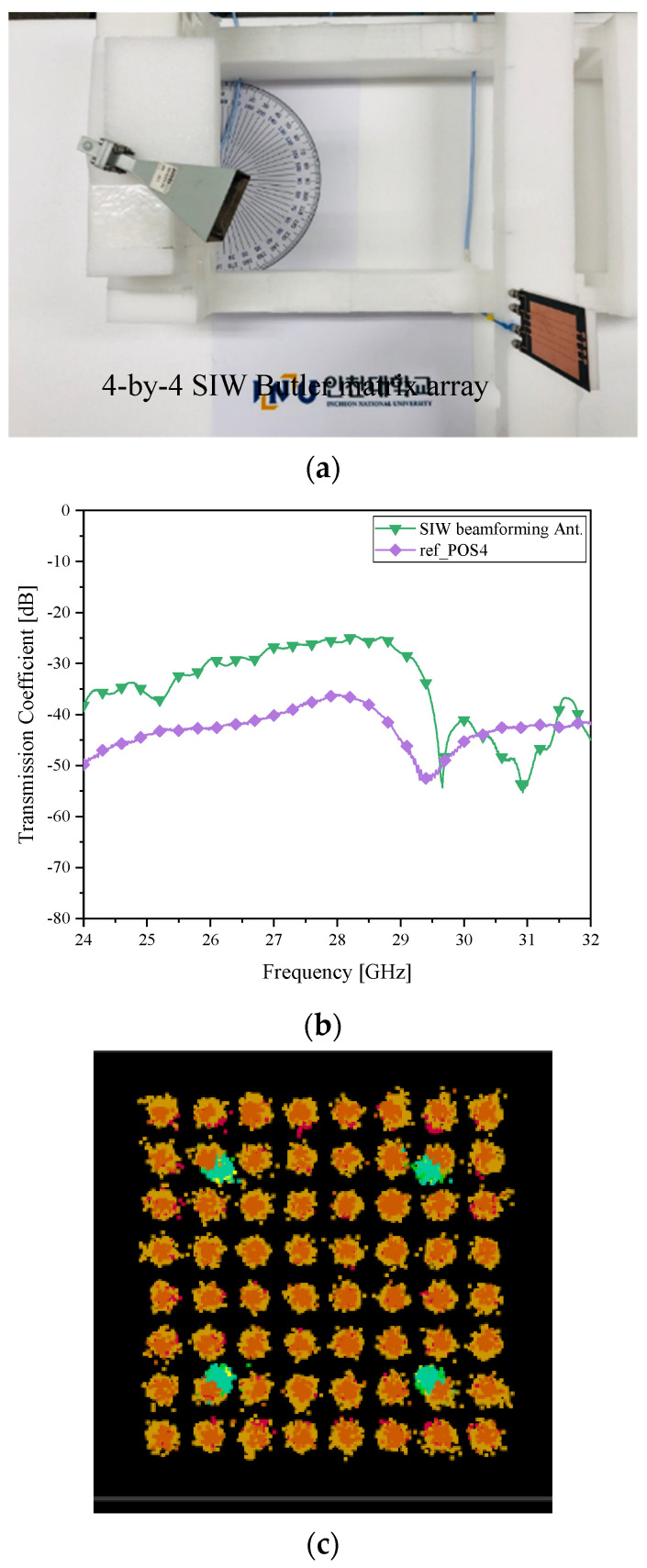
Beam alignment verification as case *δ__2R_* with the SIW Butler matrix as the RX antenna (**a**) RF-to-RF connection test; (**b**) transmission coefficient (improved connectivity); (**c**) I/Q Constellation (clearer than the microstrip-line counterpart).

In Figure 14a, the TX horn is rotated by 15° downward to convey RF power to the RX relocated to the angle of −30° as case *δ__2R_*. By the RF-to-RF test, the performance of the SIW beam-tilter and beam-alignment approach are verified to achieve a good level of the received power estimated to −25 dB as in Figure 14b. It is 10 dB greater than its counterpart of the microstrip-line beamformer. The strengths of the SIW Butler matrix antenna are presented by the 64-QAM constellation in Figure 14c, which is much clearer than Figure 10c. The improvement investigated in these experiments reveals a hint that for a very high frequency such as 28 GHz or beyond, suppressing the leakage of the wireless energy in the transmission-line can make difference in the quality of mobile communication, which should be treated with handling loss from materials and high-order coupling [20].

In other’s ways to observe the functions of 5G communication, it is hard to explicitly see the roles of components in a full system [21,22,23,24,25,26,27,28,29,30]. Their antenna beams are steered by active phase-shifter chips, but this paper uses the feed-networks. As the expensive systems e.g., [23,24,25,28,29,30,31], the system-level view on the millimeter-wave structure is watched with the QAM and constellations.

## 4. Conclusions

As of the 5G mobile era and years to come, the beamforming capability of beamforming antennas in millimeter-wave bands is emphasized for high data-transmission rates and low interference. The telecommunication system is an integrated structure of a great number of components, and it may not be easy and clear to relate the properties of the beamforming antenna as an element directly to the eventual quality of the communication function by the system. An intuitive method was suggested as a novel and practical attempt to interpret the characteristics of the beamforming antenna being forwarded to the performances of the system. Specifically, the beam-tilting and steering abilities of the TX and RX antennas are dealt with. For a horn antenna such as the TX, microstrip-line Butler matrix and SIW Butler matrix were implemented as the RX for a comparative study. The VNA as the RF-to-RF test setup and TRX7200 as the 64-QAM measurement apparatus were employed to measure the received power and constellations as the product of TX-to-RX electromagnetic connectivity via the beamforming antennas. Ten cases were studied in the form of combinations varying the angles of the TX and RX antenna beams. The worst case in beam misalignment has the received power of −38 dB and the most contaminated QAM diagram. Beam alignment enhanced the RF-to-RF connectivity by at least 8 dB with the microstrip-line to 14 dB with the SIW beamformer. Accordingly, this enables the 64-QAM constellations to show very distinct I/Q symbols. In addition, the SIW Butler matrix beamforming antenna produced consistent and good wireless connectivity in beam alignment configurations against the angular change in beam-tilting, while the microstrip-line experiences uneven resulted in terms of the level of the receive power. Conclusively, a beam alignment controlled fully electronically and cooperatively between the TX and RX systems, based on this approach and observation of enabling the tilting of beams and strengthening a beam by reducing the leakage of the electric fields in the transmission-lines in the system, leads to successful communication.

## Figures and Tables

**Table 1:** The physical dimensions of the microstrip-line Butler matrix.

Parameter	Value
L_ms_	49 mm
D_ms_	51.92 mm
A_w_	3.48 mm
T_w_	0.7 mm
Antenna gap	8.1 mm

**Table 2:** The physical dimensions of the SIW Butler matrix.

Variable Name	Value
L_siw_	119.26 mm
D_siw_	56.9 mm
A_w_	3.53 mm
T_w_	5.8 mm
Antenna gap	5.6 mm

**Figure A1:**
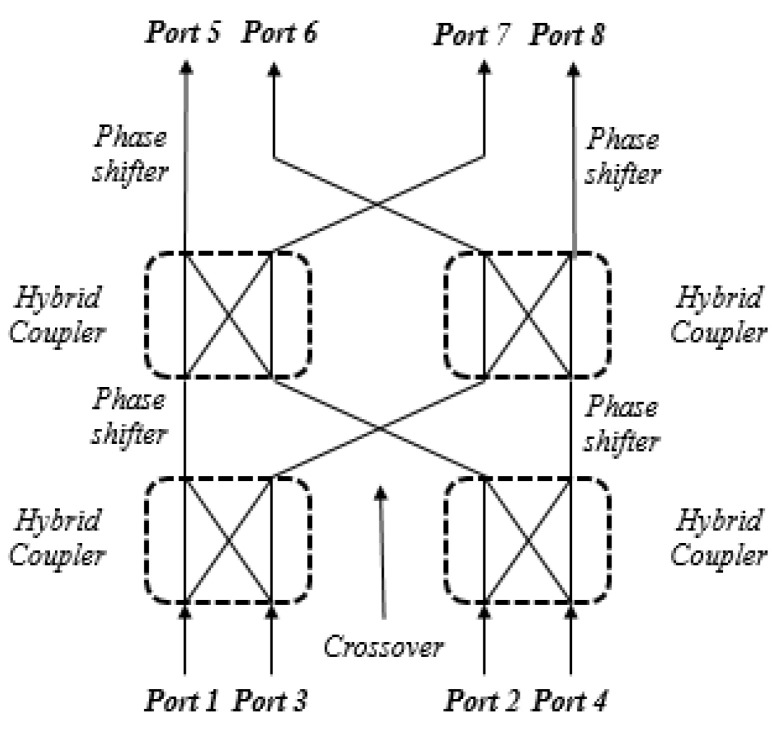
The schematic expression of the 4-by-4 Butler matrix.

**Figure A2:**
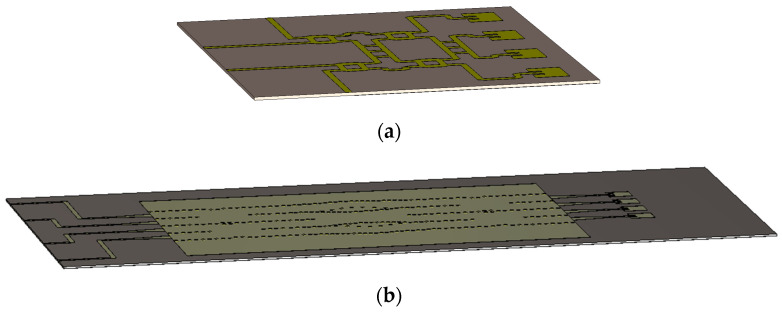
3D views of the Butler matrices (**a**) microstrip-line and (**b**) SIW Butler matrix antenna.

**Figure A3:**
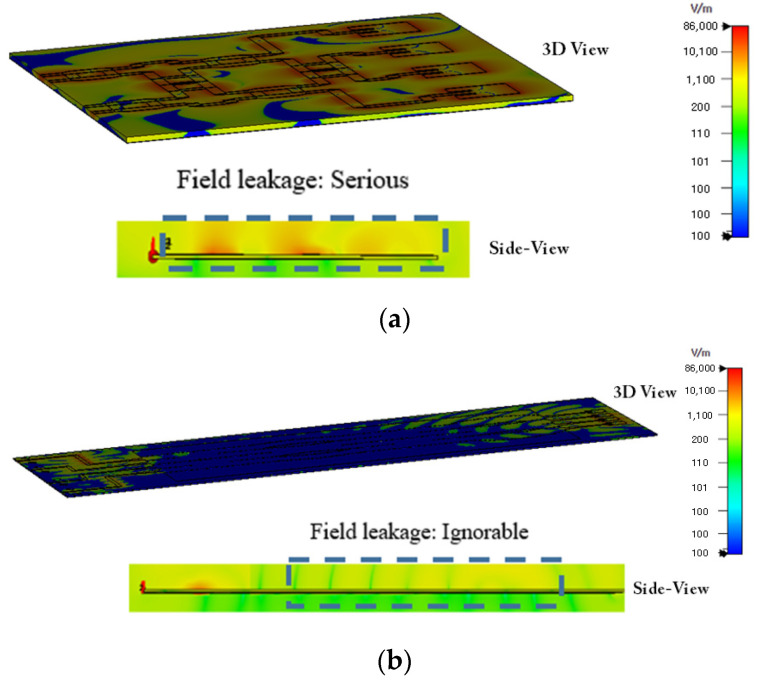
Comparing the leaked fields of the Butler matrices (**a**) microstrip-line and (**b**) SIW.

**Figure A4:**
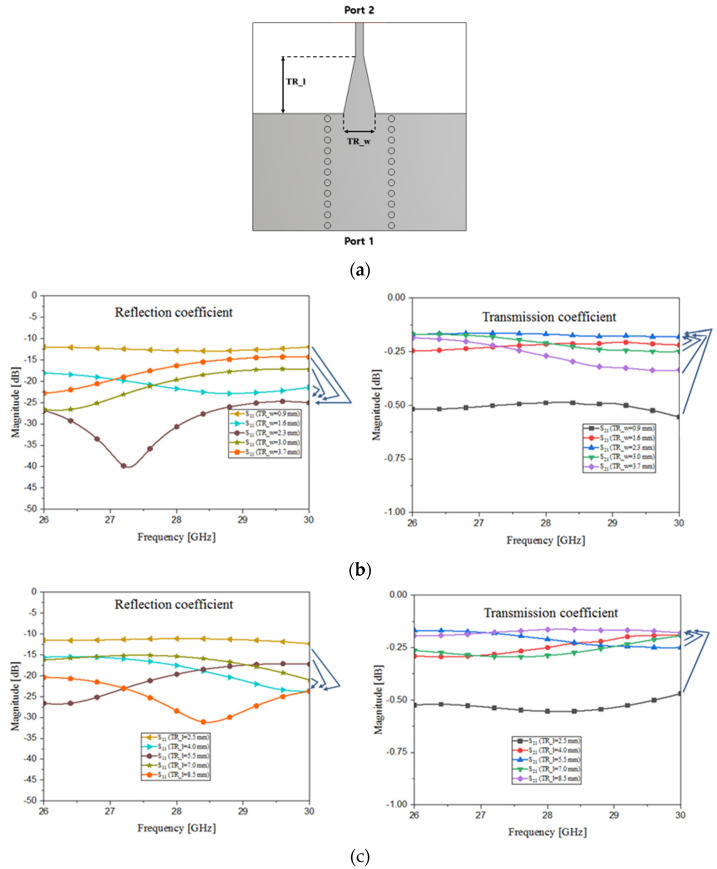
Process of finding the optimal figure of the transition of the SIW Butler matrix (**a**) geometry and parameters; (**b**) Tr_w vs. reflection and transmission coefficients; and (**c**) Tr_l vs. reflection and transmission coefficients.

## Data Availability

Not applicable.

## Appendix A

To efficiently link the TX point with the RX point in wireless communication, it is necessary to equip them with the beamforming antennas and the function of beam-tilting. To date, many technical articles have portrayed the system-level experimental configuration and performances of beamforming-based wireless link where chipsets embedded with active phase-shifters and gain-controllable power amplifiers change the angle of a beam. This ends up being costly and makes it very hard to see the quality of the mobile communication system through a subtle change in electromagnetic characteristics of the antenna. The Butler matrix antenna is suggested to meet the needs to lower the cost of development and a benefit of convenience in beam-selection. By turning on the input ports one by one using a switch, i.e., SP4T instead of the pricy phase-shifter chipset, the antenna emits the beam as in Figure 1 and Figure 2. The schematic of the Butler matrix is drawn as follows:

Figure A1 is the transmission-line feed-network made up of hybrid-couplers, cross-overs, and phase-shifters all as passive parts. Selecting one from ports 1, 2, 3, and 4 produces sets of phase distribution at ports 5, 6, 7 and 8, proper for the beams presented in Section 2.

**Table A1:** Phases at the outputs of the microstrip-line and SIW Butler matrices.

	Port 1	Port 2	Port 3	Port 4
Port 5	90°	135°	90°	135°
Port 6	−135°	90°	−135°	0°
Port 7	0°	45°	0°	−135
Port 8	135°	0°	135°	90°

The relationships of the phases at the output ports and the input ports can be understood as the selection of port 1 generating 90° at port 5, −135° at port 6, 0° at port 7, and 135° at port 8; the selection of port 2 generating 135° at port 5, 90° at port 6, 45° at port 7, and 0° at port 8; the selection of port 3 generating 90° at port 5, −135° at port 6, 0° at port 7, and 135° at port 8; and the selection of port 4 generating 135° at port 5, 0° at port 6, −135° at port 7, and 90° at port 8 as shown in Table A1. This feed-network is realized as the microstrip-line structure as something conventionally popular or the SIW as something relatively new [20,21].

Referring to geometries in Figure 1 and Figure 2 as the top views, the two structures are built in the electromagnetic simulator for design and analysis, as shown in Figure A2. The microstrip-line Butler matrix has the hybrid couplers, cross-overs, and phase-shifters exposed to the air; however, what appears from the SIW Butler matrix are the lines of tops of vias, and the passive parts are hidden from the outside, since the SIW is a type of waveguide. That is to say, the microstrip-line is a quasi-open structure, but the SIW is a quasi-closed structure. For the millimeter-wave frequency, care must be taken of minimizing the leakage of electromagnetic energy of the RF signal along the path from the input port to the radiating element for keeping the strength of radiation. To show that a popular choice from an easy design can lead to poor performances and the treatment of the energy leak really matters with regard to millimeter-wave antenna designs, the microstrip-line and SIW Butler matrix antennas were implemented and compared to each other. When Figure 7 and Figure 11 as well as Figure 1 and Figure 2 are taken for comparison, the SIW Butler matrix works much better than the microstrip-line Butler matrix. This mainly results from the ability of suppressing the leakage of the electromagnetic field from the paths from the input ports to the radiating elements.

Figure A3 highlights the advantages of the SIW over the microstrip-line beamforming antenna. The leakage of the electric field from the surface of the microstrip-line Butler matrix to the immediate upper space is severe in terms of the color code, but the dark color on the surface of the SIW implies the energy leak is ignorable. This kind of investigation has not been given in other Butler matrix antenna designs. The SIW Butler matrix in this paper is formed by monitoring the near-field leakage and far-field beam-patterns from the stage of assembling the separately designed parts and finding the optimal width and length of the transition between the output port and the radiating element to minimize the field leakage due to the reflection from the impedance mismatch at the junctions before and after the transition. These are the technical contribution factors compared to the other Butler matrix beamforming antennas. Mentioning the effects of the leakage continuously, the severe level of RF energy leak from the feed-network causes the degradation in the far-field radiation, such as a larger deviation from the desired angle for a beam. This is demonstrated in the following table:

**Table A2:** Error between the simulated and measured beams related to Figure 1 and Figure 2.

Error	Port 1	Port 2	Port 3	Port 4
Microstrip-line	8°	0°	1°	8°
SIW	3°	0°	1°	1°

Checking the beam-patterns plotted in Figure 1 and Figure 2, as for the microstrip-line Butler matrix antenna, the maximum deviation between the simulated and measured angles for a beam is 8°. Meanwhile, as for the SIW beamformer, the error is assessed up to 3° as given in Table A2. The fact the SIW beamforming antenna outdoes the microstrip-line antenna is a reason that the communication system-level tests, e.g., 64-QAM constellation plots that have high clarity. As the contributing factors were mentioned above, the following presents the process of finding the optimal values of the geometrical parameters for the transition between the output port and the antenna element.

Figure A4a was devised to lower the reflected field at the junction between the output of the Butler matrix and the radiating element. It should be optimized to guarantee the shortcomings related to the field leak due to the reflection. Tr_w as the width of the transition is found as it varies from 0.9 to 3.7 mm and obtaining the low reflection coefficient and high transmission coefficient. Tr_l is the length of the transition. As it varies from 2.5 to 8.5 mm, the value is decided with the low reflection coefficient and high transmission coefficient. Lastly, the features of the proposed methodology on the performances of 5G wireless connectivity and beamforming are addressed in comparison with the latest 5G mobile communication systems.

**Table A3:** Comparing the proposed methodology and other communication systems.

	Direct RF-to-RF Link Test of the Tx and Rx Antennas S_21_ (Port-to-Port) of VNA (Component-Level Perspective)	Treating the Angles of the Tx and Rx Beams (Aligning the Tx and Rx Beams) (Component-Level Perspective)	Connectivity Test (Communication-System-Level Perspective)		Using the Chipset			Frequency (*f* > 6 GHz?)	Beamforming Antenna (Designed or Procured)
			QAM	Constellation	Active Phase Shifter	Active Power amp.	Remarks, If Any		
[22]	ⅹ	ⅹ	ⅹ	ⅹ	O	O		15 GHz	Procured
[23]	ⅹ	ⅹ	O (16-QAM)	O	O	O	Bi- CMOS	28 GHz	Patch array (Designed)
[24]	ⅹ	ⅹ	O (64-QAM)	O	O	O	CMOS	28 GHz	Yagi array (Designed)
[25]	ⅹ	ⅹ	O (256-QAM)	O	O	O	LTCC	39 GHz	Procured
[26]	ⅹ	ⅹ	ⅹ	ⅹ	O	O		28 GHz	Procured
[27]	ⅹ	ⅹ	ⅹ	ⅹ	O	O		28 GHz	Patch array (Designed)
[28]	ⅹ	ⅹ	O (64-QAM)	O	O	O	MOSFET	39 GHz	Patch array (Designed)
[29]	ⅹ	ⅹ	O (256-QAM	O	O	O		2.4 GHz	Dipole array (Procured)
[30]	ⅹ	ⅹ	O (64-QAM)	O	O	O	GaAs	39 GHz	Yagi array (Designed)
[31]	ⅹ	ⅹ	O (64-QAM)	ⅹ	O	O		3.5 GHz	Horn (Procured)
This work	O	O	O (64-QAM)	O	ⅹ	ⅹ		28 GHz	Patch array (Designed)

As presented in Table A3, surveying the articles reporting the recent communication systems and link test results, the representative characteristics are addressed with the questions such as Direct RF-to-RF link test of the Tx and Rx antennas S21 (port-to-port) of VNA(component-level perspective), treating the angles of the Tx and Rx beams (aligning the Tx and Rx beams)(component-level perspective), connectivity test(communication-system-level perspective), using the chipset, frequency (*f* > 6 GHz?) and beamforming antenna (designed or procured). Most of them speak about the development of 5G communication systems using the chipsets and power amplifiers, while this work adopts the passive components. It is a matter of fact that for the 64-QAM tests, a testing apparatus with the up/down-converter was used, but it is to present the correlations with electromagnetic properties and beams of the antenna. This approach is quick and handy for providing the antenna developers with intuitions on the functions of the subsequent integration to a wireless system.
